# Placental imprinting: Emerging mechanisms and functions

**DOI:** 10.1371/journal.pgen.1008709

**Published:** 2020-04-23

**Authors:** Courtney W. Hanna

**Affiliations:** 1 Centre for Trophoblast Research, Department of Physiology, Development, and Neuroscience, University of Cambridge, Cambridge, United Kingdom; 2 Epigenetics Programme, Babraham Institute, Cambridge, United Kingdom; University of Pennsylvania, UNITED STATES

## Abstract

As the maternal–foetal interface, the placenta is essential for the establishment and progression of healthy pregnancy, regulating both foetal growth and maternal adaptation to pregnancy. The evolution and functional importance of genomic imprinting are inextricably linked to mammalian placentation. Recent technological advances in mapping and manipulating the epigenome in embryogenesis in mouse models have revealed novel mechanisms regulating genomic imprinting in placental trophoblast, the physiological implications of which are only just beginning to be explored. This review will highlight important recent discoveries and exciting new directions in the study of placental imprinting.

## Author summary

The placenta is essential for healthy pregnancy because it supports the growth of the baby, helps the mother’s body adapt, and provides a connection between mother and the developing baby. Studying gene regulation and the early steps in placental development is challenging in human pregnancy, so mouse models have been key in building our understanding of these processes. In particular, these studies have identified a subset of genes that are essential for placentation, termed imprinted genes. Imprinted genes are those that are expressed from only one copy, depending on whether they were inherited from mom or dad. In this review, I describe recent novel approaches used to study the mechanisms regulating these imprinted genes in mouse models, and I highlight several new discoveries. It has become apparent that the regulation of imprinted genes in placenta is often unique from other tissues and that there are species-specific mechanisms allowing the evolution of new imprinted genes specifically in the placenta.

## The link between placentation and genomic imprinting

The cells that make up the placenta perform a diversity of functions in pregnancy, including invasion into the maternal uterus, remodelling maternal vasculature, mediating nutrient and waste exchange between mother and foetus, producing pregnancy-supporting hormones, and modulating the maternal immune system to tolerate and support pregnancy. Although the placenta primarily comprises cell types that arise from the conceptus, maternal immune and endometrial cells also contribute to its development and differentiation [1–3]. Signals from the placenta modulate foetal growth and development, as well as maternal physiology. It is through these selective pressures that genomic imprinting is thought to have evolved in placental mammals, with the parental alleles in the foetal genome competing to influence maternal resource allocation [4].

Imprinted genes are those that are expressed monoallelically based on parent of origin, and over 100 imprinted genes have been identified to date in mice and humans [5], a number of which have been shown to be essential for foetal growth, placentation, and/or neurological function [6]. Furthermore, the tissues that show imprinted expression of the highest number of genes are the placenta and brain [5]. Given the developmental importance and evolutionary link between genomic imprinting and the placenta, a growing body of work has centred on investigating the regulatory mechanisms and function of imprinted genes in the placenta. In particular, the genetic tools and access to early embryonic stages in mouse models and recent advances in low-input sequence methodologies have led to several exciting new discoveries. In this review, I will discuss recent work that has revealed unique mechanisms of imprinting in the murine placenta, the role these genes play in pregnancy, and the aspects we still do not understand, including whether analogous mechanisms exist in the human placenta.

## Regulation of genomic imprinting by DNA methylation

Differentially methylated regions (DMRs) inherited from the germline typically regulate the monoallelic expression of imprinted genes. In total, 24 imprinted germline DMRs (gDMRs) have been identified in the mouse genome: 21 maternal gDMRs inherited from the oocyte and three paternal gDMRs inherited from sperm [7]. Many of these imprinted gDMRs act as imprinting control regions (ICRs) regulating the monoallelic expression of not just one gene but clusters of genes. The majority of ICRs directly regulate a promoter for either a messenger RNA or a long noncoding RNA (lncRNA) through the silencing of one allele by DNA methylation. However, the imprinting of gene clusters often involves more elaborate molecular mechanisms, such as (1) transcriptional disruption by an antisense gene [8], (2) differential targeting of enhancer/insulator elements [9], (3) altered use of polyadenylation signals [10], and (4) allele-specific targeting of epigenetic modifiers [11]. The detailed investigation of the regulatory mechanisms at imprinted gene clusters has provided invaluable insights into the epigenetic regulation of gene expression because these provide definitive examples of epigenetic states preceding transcriptional states.

The investigation of imprinted genes using next-generation sequencing techniques necessitates distinguishing the maternal and paternal alleles. Two main approaches have been employed in mouse models to facilitate this, including the comparison of reconstituted embryos with only either maternal DNA (parthenogenetic or gynogenetic) or paternal DNA (androgenetic) [12,13] and the use of allele-specific single nucleotide polymorphisms (SNPs) in F1 hybrid mice from distantly related inbred strains [14]. In combination with gene-targeting approaches, the study of genomic imprinting in mice has proven to be powerful in elucidating the mechanisms involved in setting, maintaining, and resetting imprinted epigenetic marks in development.

After genome-wide erasure in primordial germ cells, DNA methylation is reestablished during gametogenesis through the differential targeting of de novo DNA methyltransferase 3A (DNMT3A) and the essential catalytically inactive cofactor DNMT3L [15,16]. In oocytes, DNA methylation is almost exclusively targeted to transcribed gene bodies [17], whereas in sperm, the majority of the genome becomes highly methylated, with the exception of a subset of regulatory elements and cytosine (C) and guanine (G)-rich regions of the genome, termed CpG islands. There are several thousand CpG islands that are differentially methylated between oocyte and sperm; however, the vast majority lose their differential marking during epigenetic reprogramming events in early embryogenesis [18,19]. After fertilisation, the paternal genome is actively demethylated, whereas the maternal genome is predominantly passively demethylated until the blastocyst stage, at which time only a small fraction of genomic methylation is still present [20]. Imprinted gDMRs in particular are protected from these erasure events by recruiting maintenance DNMT1 through the recognition of a methylated sequence motif by zinc-finger proteins ZFP57 and ZFP445, along with the tripartite motif containing 28 (TRIM28, also known as KAP1) protein [21–23].

After the embryo implants into the maternal uterus and initiates gastrulation, there is genome-wide reacquisition of DNA methylation through the targeting of de novo DNMTs: DNMT3A and DNMT3B [24]. Notably, during this postimplantation epigenetic programming, the genome in embryonic tissues becomes highly methylated, whereas extraembryonic tissues acquire an atypical partially methylated state [25]. Despite these global differences, imprinted gDMRs maintain their allele-specific DNA methylation patterning in both lineages through protection of the unmethylated allele [26], although the mechanisms underlying this protection remain unclear.

Imprinted secondary DMRs (sDMRs) are regions that acquire differential DNA methylation during embryonic development rather than inheriting it from the germline. The majority of sDMRs identified to date are located distal to and regulated by a gDMR [27]. Nevertheless, the mechanisms regulating the establishment of sDMRs are comparatively understudied. One mechanism that recurs across several imprinted loci is the presence of a gDMR-regulated monoallelic transcript spanning a promoter and/or CpG island [27]. Consequently, DNMT3B is targeted to sites of transcriptional elongation [28], resulting in the acquisition of DNA methylation along the transcribed allele. However, as many sDMRs are not located within transcribed regions, there must be alternative mechanisms targeting allelic de novo DNA methylation at these loci. Although it has been demonstrated that the differential DNA methylation at sDMRs is important for imprinted gene regulation, such as cyclin-dependent kinase inhibitor 1C (*Cdkn1c*) [29], it remains untested at the majority of domains.

**Fig 1:**
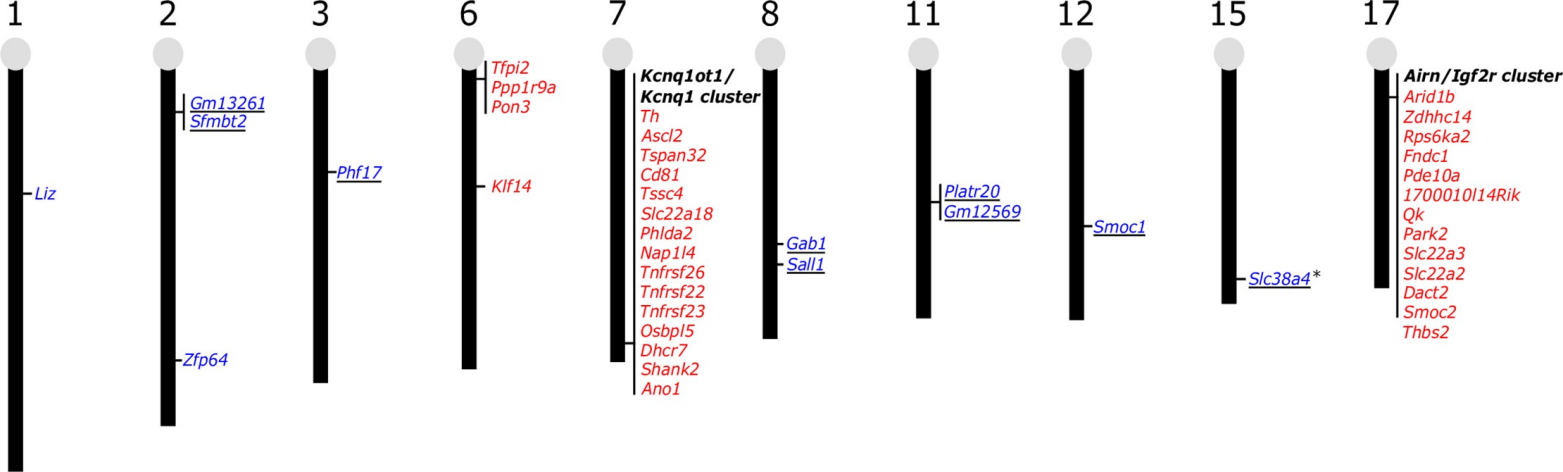
Extraembryonic-specific imprinted genes in the mouse genome. Genes reported to show imprinted gene expression almost exclusively in placenta and/or visceral endoderm [5,33,35–40]. Red genes are maternally expressed, whereas blue genes are paternally expressed. Genes that are noncanonically imprinted are underlined. Asterisks mark genes that are imprinted by an alternative mechanism in somatic tissues.

The advent of novel methods in low-input high-throughput sequencing has enabled the study of epigenetic modifications in germ cells and early embryos, providing important insights into dynamics and regulation of imprinted domains in mouse models. These advances have brought back into focus a finding that was reported early in the imprinting field: the epigenetic regulation of many imprinted loci is unique in the extraembryonic compared with embryonic lineages [30–33]. Among the most well-characterised examples is imprinted X chromosome inactivation (XCI) in extraembryonic tissues, which has been reviewed comprehensively elsewhere [34]. In this review, I will discuss autosomal loci in the mouse genome that also demonstrate that the ‘reading’ and regulation of inherited epigenetic marks between embryonic and extraembryonic lineages differ, resulting in a large number of extraembryonic-specific imprinted genes (**Fig 1**).

## Novel mechanisms of imprinting in placenta

The identification and validation of placental-specific imprinted genes has been a challenging endeavour. Allelic RNA sequencing approaches face several confounds: (1) allelic gene expression at a given time point is not comprehensive, because not all genes are expressed highly enough for allelic evaluation; (2) depending on the F1 hybrid cross, analysis is limited to a subset of genes containing at least one (but preferably more) strain-specific coding SNP; (3) in the assessment of differentiated placental tissues, mixed cell populations may obscure cell type–specific imprinting; and (4) ‘contaminating’ maternal immune and decidual cells in placental tissues can result in false positives [38]. Thus, complementary studies using in vitro trophoblast stem cells, gene-targeting approaches, and epigenomic sequencing of in vivo F1 hybrid tissues are essential to validate candidate loci and explore mechanisms of epigenetic regulation.

### Secondary imprints

**Fig 2:**
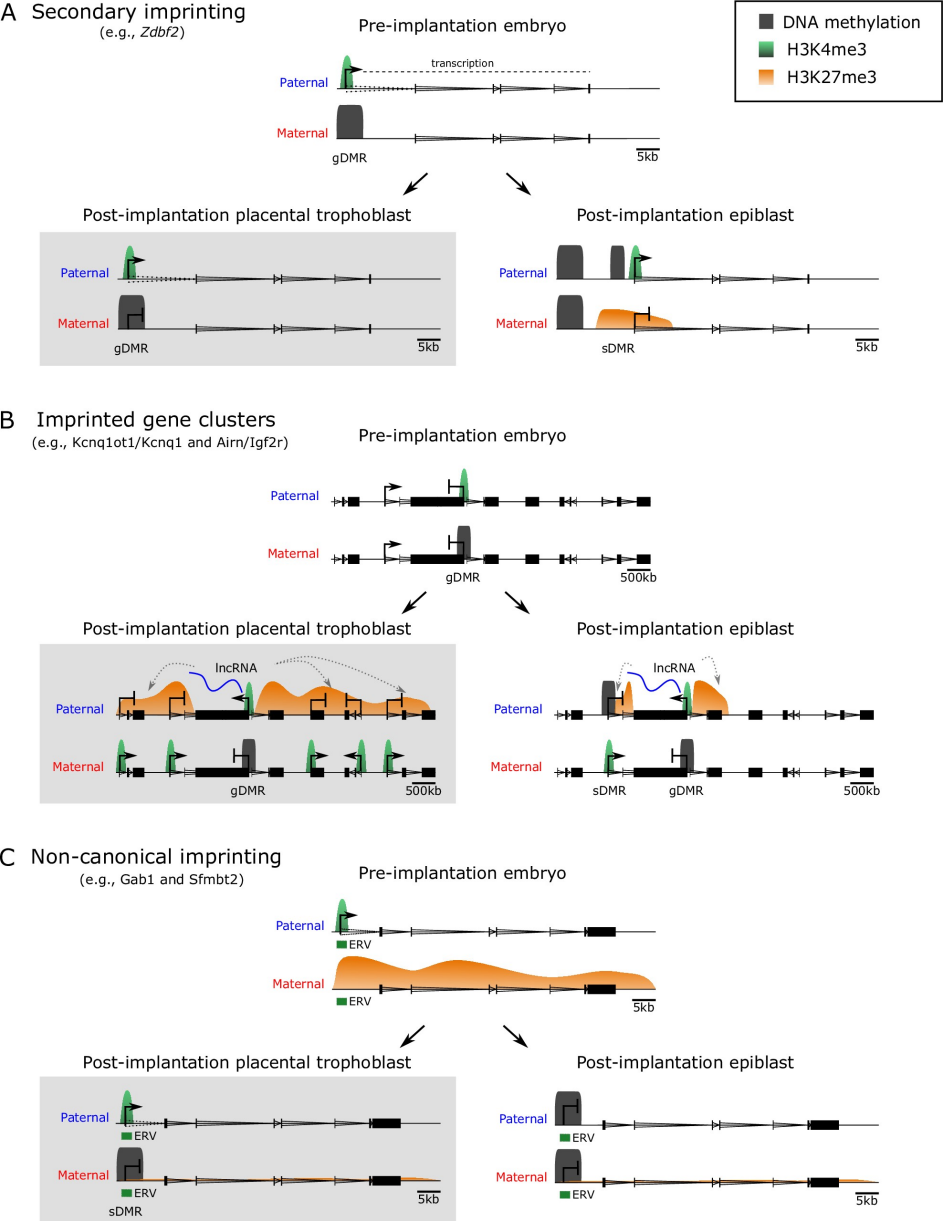
Novel mechanisms of imprinted gene regulation in placenta. There are several examples of imprinted loci that acquire different epigenetic patterning in postimplantation development between the placental trophoblast and epiblast: (A) Secondary imprinting, such as the *Zdbf2* locus. Transient monoallelic transcription of the paternal *Liz* occurs in the preimplantation embryo, with the maternal allele being silenced by a gDMR. Because of traversing transcription, the epigenetic status of the paternal allele is remodelled in the epiblast, acquiring DNA methylation at the downstream sDMR and active H3K4me3 at the *Zdbf2* promoter. Therefore, even though the maternal gDMR is reprogrammed and loses its imprinted status in the epiblast, *Zdbf2* retains imprinted expression. In trophoblast, the maternal gDMR persists, and *Liz* continues to be paternally transcribed. (B) Large imprinted gene clusters, such as the *Kcnq1ot1/Kcnq1* or *Airn/Igf2r* loci. A maternal gDMR silences the expression of an lncRNA, resulting in monoallelic expression from the paternal allele. The lncRNA associates with polycomb group proteins and potentially other epigenetic modifiers and/or repressive complexes, resulting in deposition of H3K27me3 along the paternal allele. In the epiblast, genes in relatively close proximity to the gDMR are silenced on the paternal allele because of acquisition of sDMRs at their respective promoters. In trophoblast, the silencing on the paternal allele is expansive, with H3K27me3 spreading along the paternal chromosome, silencing genes megabases away. (C) Noncanonical imprinting, such as *Gab1* and *Sfmbt2* loci. Maternally inherited H3K27me3 silences the expression of ERVs, which become actively transcribed on the paternal allele in the preimplantation embryo. In the postimplantation epiblast, these ERVs are silenced by DNA methylation, resulting in a loss of imprinting in somatic lineages. Although maternal H3K27me3 is lost, DNA methylation is acquired on the maternal allele in trophoblast, resulting in the generation of an sDMR. Hence, monoallelic paternal expression of noncanonically imprinted ERVs is maintained in trophoblast, which can confer imprinting of nearby protein-coding genes. *Airn*, antisense of *Igf2r* nonprotein coding RNA; ERV, endogenous retrovirus; gDMR, germline differentially methylated region; H3K4me3, histone 3 lysine 4 trimethylation; *Igf2r*, insulin-like growth factor 2 receptor; *Kcnq1*, potassium voltage-gated channel subfamily Q member 1; *Kcnq1ot1*, KCNQ1 opposite strand/antisense transcript 1; *Liz*, long isoform of *Zdbf2*; lncRNA, long noncoding RNA; sDMR, secondary differentially methylated region; *Sfmbt2*, Scm like with four mbt domains 2; *Zdbf2*, zinc finger DBF-type containing 2.

Across several imprinted domains, differences in the acquisition of sDMRs between the embryonic and extraembryonic lineages have been observed [30,31,35]. In particular, the dynamics observed at the zinc finger DBF-type containing 2 (*Zdbf2*) locus on chromosome 1 highlight the distinct epigenetic regulation in these developmental lineages. *Zdbf2* is an imprinted gene with paternal expression and, paradoxically, a paternal DMR near its promoter. Early studies of *Zdbf2* suggested this locus may be a paternal gDMR [41]; however, work in preimplantation embryos showed that paternal DNA methylation was erased and reset secondarily in postimplantation development [35]. This paternal sDMR was established because of the transient monoallelic expression of a traversing long isoform of *Zbdf2* (*Liz*) originating from an upstream transcription start site, which was regulated by a maternal gDMR (**Fig 2A**) [35]. The paradoxical finding of the paternal sDMR adjacent to a paternally expressed gene was later explained through a series of experiments systematically ablating epigenetic modifiers [42]. Acquisition of DNA methylation at the sDMR was required to prevent accumulation of repressive histone 3 lysine 27 trimethylation (H3K27me3) across the paternal allele, and consequently, conferred an active chromatin state at the adjacent *Zdbf2* promoter. The interrogation of early embryonic stages was essential in capturing this mechanism because of the transient nature of *Liz* transcription and the subsequent loss of the maternal gDMR in somatic tissues.

Conversely, in extraembryonic lineages, the upstream maternal gDMR remains intact throughout the postimplantation epigenetic programming [35]. In turn, the paternal sDMR is not appropriately established, the canonical *Zdbf2* promoter remains silenced, and there is continued paternal expression of *Liz* throughout placental development (**Fig 2A**) [35]. Thus far, it remains unclear why the maternal gDMR is reprogrammed in the embryo and not in the extraembryonic tissues. Continued expression of *Liz* from the paternal allele in extraembryonic tissues may be sufficient to protect it from acquiring DNA methylation, or conversely, extraembryonic tissues may be lacking a factor that is required to silence *Liz* in the epiblast. In general, investigations into the differential targeting of sDMRs will provide valuable insights into factors required for reprogramming or preserving imprinted epigenetic marks through postimplantation development.

### Imprinted gene clusters

Although many imprinted genes are located in clusters, two imprinted gene clusters contain a subset of genes with imprinted expression only in the placenta: the KCNQ1 opposite strand/antisense transcript 1(*Kcnq1ot1*)/potassium voltage-gated channel subfamily Q member 1 (*Kcnq1*) cluster on chromosome 7 and antisense of IGF2R nonprotein coding RNA (*Airn*)/insulin like growth factor 2 receptor (*Igf2r*) cluster on chromosome 17. These imprinted domains each contain a maternal gDMR that acts as an ICR. In both examples, the maternal gDMR directly represses the expression of an lncRNA (*Kcnq1ot1* and *Airn*, respectively) such that it is only expressed from the paternal allele. In turn, the expression of paternal *Kcnq1ot1* and *Airn* acts to silence overlapping antisense (*Kcnq1* and *Igf2r*, respectively) and distal protein-coding genes *in cis*, meaning on the same chromosome from which they were transcribed [43,44]. In somatic tissues, the extent of imprinting is restricted to a ‘core’ subset of genes within relatively close proximity to the gDMRs. However, in the placenta, numerous genes, as far away as 7.7 megabases (Mb), are silenced on the paternal allele (**Fig 2B**) [36,37,39,43,45]. To date, placental-specific imprinted gene expression has been observed for 15 genes distal of *Kcnq1ot1/Kcnq1* and 12 genes distal of *Airn/Igf2r* (**Fig 1**). The difference in the extent of imprinting within these clusters in somatic tissues and placenta has been an area of ongoing research and is still not entirely understood.

The placental-specific repression of genes along the paternal allele of *Kcnq1ot1/Kcnq1* and *Airn/Igf2r* clusters was first linked to histone modifications in the early characterisations of knockout mouse models. Ablation of embryonic ectoderm development (EED), a component of the polycomb repressor complex 2 (PRC2) that deposits H3K27me3, and euchromatic histone lysine methyltransferase 2 (EHMT2, also known as G9A), which deposits H3K9me2, showed a loss of imprinting of distal genes in the *Kcnq1ot1/Kcnq1* cluster [46–49]. Detailed characterisation of the *Kcnq1ot1/Kcnq1* and *Airn/Igf2r* clusters in placental trophoblast demonstrated that PRC1 and PRC2 localisation and H3K27me3 were targeted to the locus *in cis* by the paternally expressed lncRNAs [11,30,50]. However, why not all genes along the paternal allele are imprinted is still unclear, although recent work has made several advances in revealing the underlying mechanisms.

A targeted deletion of the entire *Airn* gene indicated that the *Airn* lncRNA, rather than its genomic features, was essential for allelic silencing of nonoverlapped genes on the paternal allele [45]. To understand how lncRNAs could target such large genomic regions, the roles of chromosome folding, lncRNA abundance, and CpG islands in the targeting of *Airn* and *Kcnq1ot1* lncRNAs was assessed in trophoblast stem cells [51]. The authors found that chromosome folding brought distal regions into close proximity of the transcribed lncRNA on the paternal allele [51], consistent with allelic differences in folding of the *Airn/Igf2r* locus seen in vivo [45]. CpG islands, in particular, enabled PRC targeting and spreading along the silenced paternal alleles within these imprinted domains [51]. However, the 3D folding of these regions and the proportion of polycomb-bound CpG islands were similar between embryonic stem cells and trophoblast stem cells. Furthermore, despite an almost 10-fold difference in size between the *Airn/Igf2r* and *Kcnq1ot1/Kcnq1* imprinted domains, the lncRNAs were expressed to similar levels and had a similar half-life. Thus, it appears that neither chromosome conformation, polycomb-bound CpG islands, nor the level of lncRNA expression can explain why these H3K27me3-repressed imprinted domains differ in size across domains or developmental lineages.

There are several remaining possible mechanisms that could explain why lncRNAs exert different activity in extraembryonic versus embryonic lineages: (1) differential expression and/or posttranslational modifications of epigenetic modifiers and cofactors could alter their recruitment to these loci, (2) altered temporal expression of lncRNAs during development could alter their silencing capacity, or (3) the hypomethylated state of DNA in extraembryonic tissues may permit spreading of repressive histone modifications that is not permitted in the highly methylated embryo. Determining which of these mechanisms is at play will be valuable in understanding in general how lncRNAs are targeted to and can modulate chromatin accessibility.

### Noncanonical imprinting

Although it was postulated that inherited histone modifications may be able to regulate imprinted expression, the first such gDMR-independent (termed ‘noncanonical’) imprinted genes were only recently definitively identified [33]. By comparing regions of open chromatin between gynogenetic and androgenetic embryos, Inoue and colleagues identified several paternally expressed imprinted genes that were not associated with maternal gDMRs but, rather, maternal H3K27me3. Through the injection of an H3K27-demethylase in early embryos [33], and later using a conditional knockout for PRC2 component EED in oocytes [52], they were able to demonstrate that H3K27me3 inherited from the oocyte was required to silence the maternal allele of noncanonically imprinted genes. In a complementary approach using a conditional knockout for DNMTs in oocytes, imprinted regulation of these genes was shown to be DNA methylation independent, confirming that these regions are truly noncanonical in nature [32,38,39]. Although most noncanonical imprinted gene expression appeared to be transiently present in the early embryo [33], in extraembryonic lineages (i.e., yolk sac and placenta), there were a handful of genes for which imprinted monoallelic expression persisted into the later development [32,33,38,39].

The mechanisms underlying this lineage-specific imprinting are only beginning to be uncovered. Notably, a number of noncanonically imprinted domains, identified as monoallelic H3K4me3 peaks, overlapped endogenous retroviral (ERVs) insertions, many of which were sites of imprinted transcription initiation [39]. These noncanonically imprinted ERVs acted as alternative promoters for nearby protein-coding genes (e.g., GRB2-associated binding protein 1 [*Gab1*], SPARC-related modular calcium binding 1 [*Smoc1*]) or drove expression of noncoding RNAs. It remains to be tested whether a subset of these imprinted ERVs can also function as enhancers for nearby genes or whether the noncoding RNAs themselves are functionally significant.

Given the lineage-specific nature of noncanonical imprinted gene expression, it is not surprising that the epigenetic status of noncanonical imprinted domains differs in embryonic and extraembryonic postimplantation development. Initially these regions inherit H3K27me3 from the oocyte on the maternal allele, but this repressive modification is gradually lost in preimplantation development, along with the majority of genomic H3K27me3 (**Fig 2C**) [39,53]. In postimplantation epigenetic programming, the maternal allele becomes DNA methylated in the extraembryonic lineages creating sDMRs at the noncanonically imprinted ERVs [39]. In contrast, in the embryonic lineages, DNA methylation is acquired on both alleles, resulting in a loss of imprinting in somatic tissues. The requirement of allelic DNA methylation for regulating the associated imprinted gene expression of ERVs has yet to be tested. At present, it is still not understood why noncanonical imprints transition to sDMRs rather than maintain/reestablish allelic H3K27me3. Furthermore, the acquisition of allelic DNA methylation does not appear to be associated with a DMR-spanning transcript, suggesting that unknown factors are important for protecting the active allele and/or demarking the silent allele for DNA methylation in extraembryonic lineages.

## Placental-specific imprinting: A role for ERVs

The emergence of imprinting has been repeatedly linked to retrotransposon insertions [54]. This appears to be particularly true in the germline, in which transcriptionally active ERVs drive expression of novel transcripts conferring DNA methylation in the oocyte [55] or are the target of piwi-RNA silencing mechanisms in sperm [56]. Placental-specific imprinted genes and gDMRs appear to be largely species specific in their imprinting status [38,57], supporting that placental-specific imprinting may have recently evolved imprinted expression and/or epigenetic modifications. Evidence supports a role for ERVs in this evolution of imprinting, such that species-specific ERV insertions transcribed in oocytes can generate species-specific maternal gDMRs, several of which only retain their imprinting status in the trophectoderm during embryogenesis, becoming placental-specific imprinted gDMRs [57]. The genome-wide hypomethylation in the placenta may also present a unique opportunity for the acquisition of novel regulatory roles of ERVs. Yet unlike in the germline, in which ERVs influence the deposition of imprints, active ERVs in the placenta may permit the persistence of these inherited allelic epigenetic marks, which would otherwise be reprogrammed.

A number of observations support the idea that the placenta has a uniquely permissive epigenetic landscape allowing novel forms of gene regulation by ERVs. A subset of ERVs were shown to function as enhancers in placental trophoblast through intrinsic transcription factor binding motifs and contact with gene promoters [58,59]. The syncytin genes, which are essential for the formation of the multinucleated syncytiotrophoblast in placenta, are a gene family derived directly from the retroviral envelope gene [60]. Similarly, an imprinted ERV-derived gene, retrotransposon-like 1 (*Rtl1*), is required for placental foetal capillary formation and, consequently, maternal–foetal nutrient exchange [61]. In addition to their role in driving noncanonical imprinted gene expression [39], ERVs can act as alternative promoters for nonimprinted genes in placental trophoblast [62]. The full extent of the role and mechanisms for ERVs in imprinted gene regulation in placentation have yet to be fully explored and will likely offer exciting new insights as molecular studies are undertaken across placental mammals.

These newly evolved placental-specific imprints may be merely ‘bystanders’ with no functional consequence per se [63]. Imprinting of these loci may be a consequence of opportunistic sequence composition, transcription factor motifs, and/or recruitment of epigenetic ‘reader’ proteins, resulting in the retention of inherited epigenetic modifications. However, recent work has highlighted an important functional role for at least some placental-specific imprinted genes.

## Placental-specific imprinted gene function

**Fig 3:**
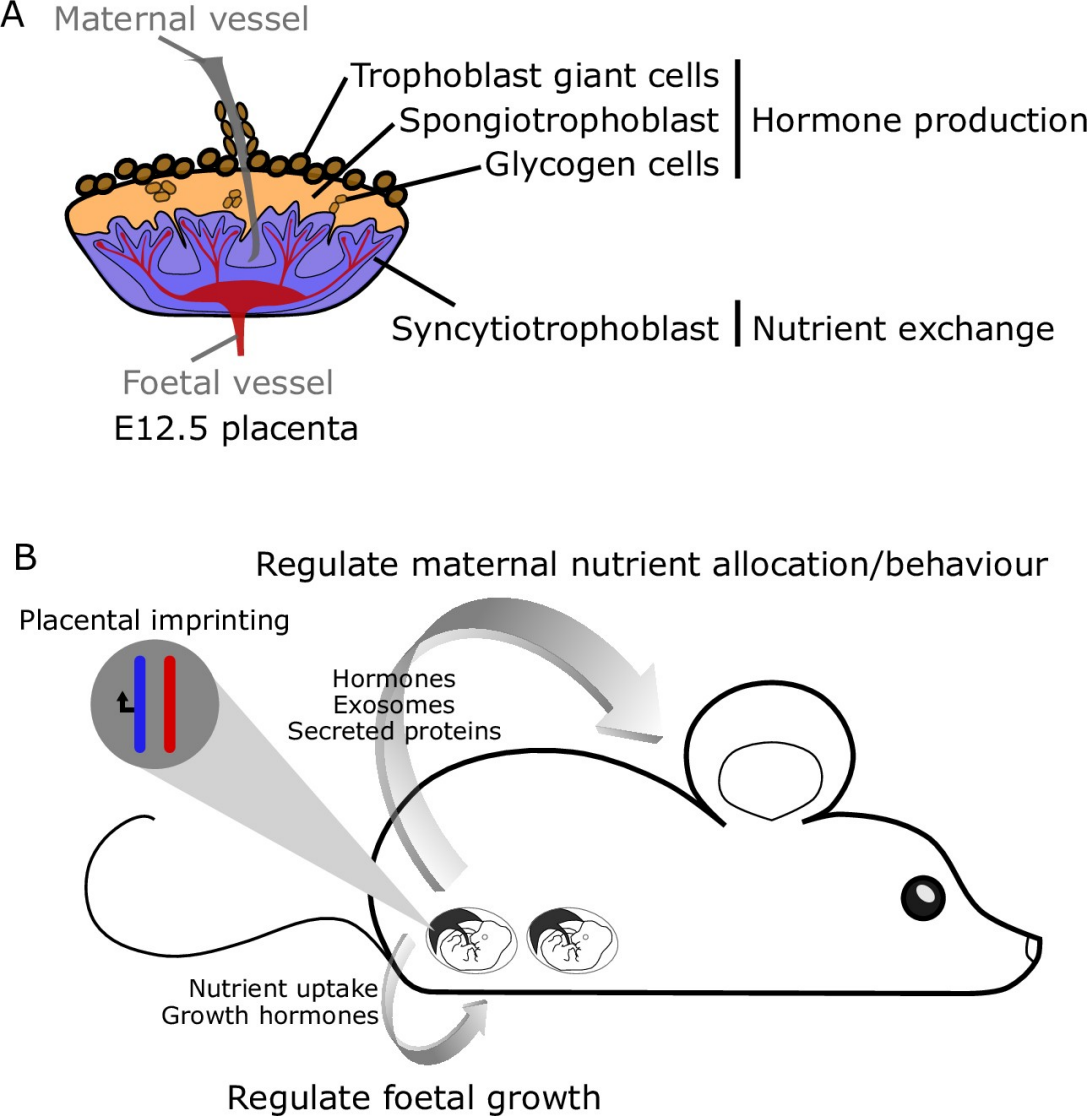
Function of placental-specific imprinting. (A) The localisation of trophoblast cell types in an E12.5 mouse placenta. The syncytiotrophoblast forms the labyrinth layer, in which nutrient, waste, and gas exchange occurs between maternal and foetal circulations. The spongiotrophoblast, glycogen, and trophoblast giant cells form the junctional zone, which is essential in hormone production to support foetal growth and maternal adaptations to pregnancy. (B) Placental-specific imprinted genes have the capacity to regulate both maternal and foetal physiology. The placenta releases a number of factors into maternal circulation, including exosomes, hormones, nucleic acids, and proteins, many of which have been shown to be essential in the maternal adaptations to pregnancy. In turn, placentation can directly regulate nutrient uptake, as well as through the production of growth hormones, to support foetal development. E, embryonic day.

The placenta forms the maternal–foetal interface and comprises many cell types, the majority of which are derived from trophoblast cells, a lineage that is specified in the preimplantation embryo as the trophectoderm. The mature murine placenta contains two essential layers: the labyrinth, which predominantly comprises syncytiotrophoblast, and the junctional zone, which contains spongiotrophoblast, glycogen, and trophoblast giant cells (**Fig 3A**). The syncytiotrophoblast are multinucleated cells that are the main site of nutrient exchange between the maternal and foetal circulations. There are various subtypes of trophoblast giant cells, which are collectively required for invasion into the maternal uterus, remodelling of the maternal vasculature, and secreting hormone and paracrine factors [64]. Together, the spongiotrophoblast, glycogen cells, and all subtypes of trophoblast giant cells make up the endocrine cells of the placenta, mediating maternal–foetal cross talk and producing hormones essential to maintain pregnancy. Although the role of placental-specific imprinting has not been studied extensively, there are some key examples that demonstrate the importance of imprinted genes in regulating not only foetal growth but also maternal nutrient allocation and behaviour (**Fig 3B**).

Among the noncanonically imprinted genes, the functions of Scm-like with four mbt domains 2 (*Sfmbt2*) and *Gab1* have been studied using transgenic mouse models. In a constitutive knockout model, *Gab1* was shown to be essential for development of the heart, placenta, and skin, and embryos showed growth deficits after embryonic day (E) 13.5 [65]. In the placenta of *Gab1* −/− embryos, the development of the syncytiotrophoblast was impaired. GAB1 is involved in signalling of growth factors and cytokines, and the syncytiotrophoblast has been shown to be sensitive to growth factor impairment in vivo [66,67]. Furthermore, defective syncytiotrophoblast formation impairs the nutrient transfer between mother and foetus and, therefore, is often associated with foetal growth restriction [68].

Conversely, the *Sfmbt2* gene is almost exclusively expressed in extraembryonic tissues and is essential for the formation and maintenance of trophoblast lineages, including the derivation of trophoblast stem cells [69]. Consequently, ablation of *Sfmbt2* leads to embryonic lethality due to aberrant development of extraembryonic tissues. SFMBT2 is a polycomb group protein but appears to be only subtly involved in gene repression; rather, its primary role may be in the repression of highly repetitive regions of the genome, with strong localisation to major satellites, pericentromeres, and long interspersed nuclear elements (LINEs) [70]. Thus, SFMBT2 may be particularly important for genomic stability in trophoblast, in which repetitive regions are hypomethylated [71]. Intriguingly, the *Sfmbt2* gene also contains a large cluster of micro-RNA (miRNA) genes within intron 10, which are also imprinted in placenta [72]. Transgenic mice generated by ablating the entire miRNA cluster had an extremely underdeveloped placental junctional zone, foetal growth restriction, and higher rates of foetal demise [72].

Although knockout models are valuable in revealing gene function, to understand the importance of imprinting in regulating gene dosage, more subtle perturbations are required. The consequences of manipulating gene dosage of two placental-specific imprinted genes in the *Kcnq1ot1/Kcnq1* cluster (achaete-scute family bHLH transcription factor 2 [*Ascl2*] and pleckstrin homology like domain family A member 2 [*Phlda2*]) have been recently studied in detail. Using a mouse model carrying a large deletion near *Ascl2*, which results in its partial loss of imprinting, it was shown that impaired expression of *Ascl2* resulted in a failure to properly establish the junctional zone [73,74]. Conversely, overexpression of *Ascl2* also resulted in impaired junctional zone development and an increase in stored glycogen due to a striking misallocation of glycogen cells to the labyrinth [75]. Glycogen cells continually release glucose throughout pregnancy, supporting foetal growth, particularly in late gestation [76]. Consistently, altering gene dosage of *Ascl2* is associated with foetal growth restriction [73,75].

Using mouse models expressing two, one, or no copies of *Phlda2*, the levels of *Phlda2* expression were shown to be essential for correct formation of the spongiotrophoblast, appropriate levels of glycogen accumulation [77], and foetal growth [78]. The placenta secretes hormones, exosomes, and proteins into maternal circulation and can thus influence maternal physiology (**Fig 3B**) [79]. As a striking example of this phenomenon, the perturbations in spongiotrophoblast function due to altered *Phlda2* expression were shown to result in changes in the maternal brain and, consequently, altered postnatal care [80].

Together, these findings suggest that placental-specific imprinting is key in the regulation of nutrient acquisition and foetal growth in mouse development. Nevertheless, the functions of the majority of placental-specific imprinted genes remain unexplored, and future work may reveal novel mechanisms for imprinting in regulating placentation and development.

## Future directions in studying placental imprinting in humans

The role of placental-specific imprinting in human development and placentation is unclear and remains challenging to study because of the inaccessibility of early embryonic development and the species-specific nature of placental imprinting. A recent study identified an association between aberrant imprinting at a number of placental-specific imprinted loci and intrauterine growth restriction, suggesting that these genes may regulate foetal growth, but determining whether these changes are the cause or consequence of poor growth will require further study [81]. New technologies in culturing human trophoblast stem cells [82] and trophoblast organoids [83,84] offer new avenues into the study of placental-specific imprinting in trophoblast differentiation and function. Furthermore, the recent advances in epi-CRISPR [85–88] will specifically allow the modulation of epigenetic states and transcriptional levels at imprinted loci in trophoblast cells in vitro to study the cellular phenotypic consequences of gene dosage.

Intriguingly, widespread placental-specific imprinted DNA methylation has been observed in humans, with more than 100 maternal gDMRs identified in placental trophoblast [89–91], a feature that is not conserved in mouse. Additionally, human placental imprinting is uniquely polymorphic between individuals [89,91], which may, in part, be attributable to differences in ZFP57 and ZFP445 activity in human and mouse embryogenesis [23]. These findings suggest that the human placenta is exceptionally permissive to inherited DNA methylation from the oocyte; however, it is yet to be shown whether any imprinted loci in the human genome are regulated noncanonically. Recent studies in human preimplantation embryos suggest that H3K27me3 is reprogrammed much more rapidly than in mouse [92]; yet early allelic data suggest that at least a few loci may maintain a maternal bias in H3K27me3 to the morula stage [93]. Nevertheless, demonstrating whether noncanonical imprinting exists in humans presents further challenges for future study because heterozygous SNPs with informative parental allelic information are far rarer than in mouse hybrids, drastically limiting the number of loci that can be evaluated in any one sample. Future work will reveal the full extent of imprinting in human placenta and help elucidate whether placental-specific imprinting has a role in regulating foetal growth and maternal adaptations to pregnancy, which will be essential for our understanding of pregnancy-related pathologies.
